# Age-dependent perfume development in male orchid bees, *Euglossa imperialis*

**DOI:** 10.1242/jeb.246995

**Published:** 2024-03-22

**Authors:** Jonas Henske, Thomas Eltz

**Affiliations:** Department of Animal Ecology, Evolution and Biodiversity, Ruhr-Universität Bochum, 44801 Bochum, Germany

**Keywords:** Chemical communication, Volatiles, Chemical signal, Age indicator, Fitness, Honest signal

## Abstract

Male neotropical orchid bees concoct complex perfume blends by collecting exogenous volatiles from various sources, including orchids. These perfumes, stored in specialized hind-leg pouches and released during courtship, serve as inter-sexual signals. It has been hypothesized that male perfumes honestly indicate aspects of male fitness. If perfume traits such as quantity or complexity increase over individual lifetime, perfumes could reflect age (survival) and cumulative foraging success of males. We conducted a two-season mark–recapture study with *Euglossa imperialis* in Costa Rica, monitoring the balance of perfume uptake and expenditure over individual male lifetime. We sealed one hind-leg pouch upon initial capture, ‘freezing’ the perfume status on one side, and compared it with the other side at recapture to assess changes in perfume traits over time. Additionally, we used a novel method to estimate individual age by combining two parameters of wing degradation. Contrary to predictions, young to intermediate-aged bees had the highest quantities of perfume and the highest diversity of detected compounds. At the same time, the change in perfume between recaptures was positive (increase in amount and complexity) in young bees, whereas it was neutral to negative in older bees. Although these findings do not disprove an indicator function of male perfume, they shift the emphasis to non-cumulative fitness components such as sensory acuteness or cognitive capacity as likely targets of selection. Females preferring strong perfume signals in mates would maximize speed of foraging in offspring rather than their lifetime cumulative yield.

## Teaser

**Highlighted Article:** Male orchid bees concoct environment-derived perfumes for sexual signaling. In *Euglossa imperialis*, perfume load peaked early in male lives, suggesting that it communicates non-cumulative qualities, not age.

## INTRODUCTION

Chemical signals are widespread in sexual communication systems in the animal kingdom. In insects and other arthropods, sex pheromones have been shown to mediate mate and species recognition and thus are preserved in chemical composition through stabilizing natural selection (Roelofs, 1995; Wyatt, 2003; Symonds and Elgar, 2008). However, recent studies revealed substantial heritable variation in pheromone traits, especially in male-calling systems, suggesting that mate choice (sexual selection) is based on chemical traits (Johansson and Jones, 2007; Ruther et al., 2009; Steiger and Stökl, 2014; Chemnitz et al., 2015; Darragh et al., 2017). Consequently, chemical traits are now more commonly perceived through the same comprehensive theoretical framework that is applicable to other secondary sexual traits, such as long tails, colorful plumage or complex songs (Steiger and Stökl, 2014). Chemical equivalents of such traits could be pheromone concentration or aspects of chemical composition that are related to physiological state or age. In particular, age has been suggested to reflect fitness because old individuals have demonstrated their capacity to survive, and age-related expression of secondary sexual traits in males may allow females to select genetically superior males (Manning, 1985; Brooks and Kemp, 2001).

Sex pheromones are usually synthesized *de novo* by the sender. However, males of the neotropical orchid bees stand out for their use of exogenous volatile substances for sexual signaling (Henske et al., 2023). By visiting floral sources for the purpose of volatile collection, male orchid bees act as specific pollinators for a large number of neotropical plants, including many orchids (Vogel, 1966; Janzen, 1971; Whitten et al., 1993). Male orchid bees store and accumulate these volatiles in enlarged hind-leg pouches that facilitate the concoction of complex perfume blends (Eltz et al., 1999). The accumulated perfumes are then exposed actively during a stereotypical display that males perform in the forest understory (Eltz et al., 2005b). Female orchid bees select conspecific males based on the possession of these perfumes, which act as inter-sexual signals that transfer information during male courtship display (Henske et al., 2023). However, what exact information is encoded in the perfume signal remains unknown. Comparative studies have shown that, chemically, orchid bee perfumes are broadly species-specific (Eltz et al., 2005a), having diverged substantially even among closely related species (Zimmermann et al., 2009; Weber et al., 2016). This suggests that male perfume signals are involved in species recognition when males of different species display in the same general area (see Pokorny et al., 2017), and that perfumes evolve by natural selection. However, intraspecific individual variation in the total amount as well as in the chemical composition of perfume blends exists (Eltz et al., 2005a; Ramírez et al., 2010), suggesting that female choice and sexual selection may shape perfume traits. Male perfumes are complex signals based on species-specific preferences for certain volatiles, which are modified by experience- and environment-dependent individual choices (Eltz et al., 2005a; Pokorny et al., 2013). Making perfume likely incurs high costs to the males, which makes perfumes potential honest indicators of survival/age, foraging success, competitive strength, cognitive skills or sensory acuteness. Foraging history or age in particular may be honestly communicated if perfume traits such as amount/intensity, overall complexity or the proportion of certain compounds increase linearly over the male's lifetime. However, knowledge of how male perfumes develop over a lifetime is still very limited.

The mechanisms themselves by which male orchid bees collect volatiles and expose perfume are well studied (for detailed information on volatile uptake, see Appendix). However, little is known about the approximate quantity of volatiles collected and/or exposed over a given period of time. With regard to uptake, most natural volatiles of interest to euglossines appear to be in very short supply, so much that the exposure of an artificial chemical bait in lowland rainforest can draw impressive numbers of males in a very short time (Roubik and Hanson, 2004). Owing to the scarcity and unpredictability of volatiles in natural environments (Janzen, 1971; Ackerman, 1989; Pokorny et al., 2013), with orchids being particularly rare because of low population densities and short-lived flowers (Ackerman, 1983), perfume likely incurs substantial costs for male euglossines. For example, some natural sources provide only minor amounts of volatiles at any given time. Male *Euglossa hemichlora* collecting from a decaying log in Panama for 1–10 min had not acquired detectable amounts of the single major compound in their hind-leg pouches (see discussion in Eltz et al., 1999). Although it is unknown whether this log was an average euglossine source, it is likely that attractive volatiles are elusive and that males need to visit and collect on repeated occasions and over longer periods of time. Repeated visits over days and weeks by marked individuals have been observed at both natural (Janzen, 1981; J.H., unpublished data) and artificial (Ackerman and Montalvo, 1985; Eltz et al., 1999; Pokorny et al., 2015a) volatile sources, suggesting that the collection process itself is time consuming, even when a scarce source has been detected.

With regard to exposure, fluorescent dye experiments have demonstrated that perfume is released from pouches as a result of stereotypical leg movements (‘leg crossings’) performed during courtship display (Eltz et al., 2005b). In cage experiments, males of *Euglossa dilemma* performed these behaviors starting within days after their emergence, even without having access to volatiles, and continued to do so until the end of the experimental trial (Henske et al., 2023). Perfume-supplemented males were estimated to have released approximately two-thirds of their perfume content over 10 days of active display with no opportunity for volatile uptake (Henske et al., 2023). At the same time, passive loss of volatiles owing to evaporation from hind-leg pouches is comparatively low (Eltz et al., 2019), indicating a high storage efficiency, potentially allowing linear accumulation of volatiles over time.

**Fig. 1.**
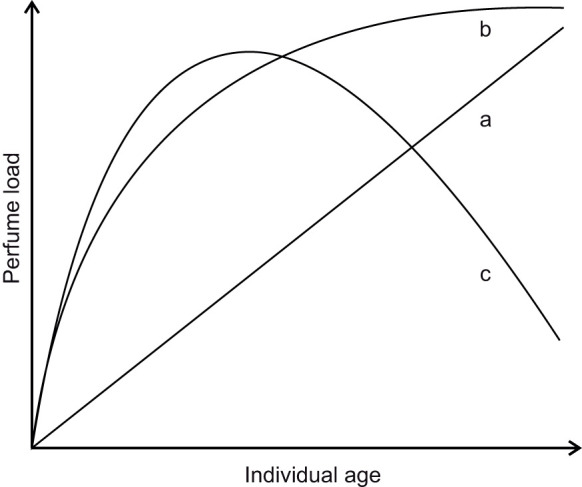
**Possible trajectories of perfume development over individual age in male orchid bees.** Perfume load (amount) is plotted, but other perfume parameters may vary in similar ways, depending on how volatile uptake (collection) and expenditure (loss/active exposure) balance out over the bee's lifetime. (a) Linear cumulative: continuous collection and continuous expenditure. (b) Saturating: declining collection and/or increasing expenditure. (c) Peaking: declining collection and increasing (heavy) expenditure.

Male orchid bees are relatively long-lived in comparison with males of many other bees. A previous study revealed adult life spans between 6 weeks to 6 months depending on the size of the species (Ackerman and Montalvo, 1985). The amount of perfumes extracted from field-caught males from a range of species showed only minor variations depending on whether the males were caught at display sites (sites of perfume exposure) or at artificial scent baits (sites of volatile uptake; Pokorny et al., 2017). Thus, volatile acquisition and expenditure appear to happen in parallel over most of the life of male orchid bees. Different scenarios based on how exactly uptake and exposure balance over individual lifetimes are possible (see Fig. 1). Knowledge of these scenarios (linear cumulative, saturating or peaking quantity/complexity of perfumes over time) would have profound consequences on what fitness components are communicated in perfume signals. Currently, our empirical insights into how perfumes are related to age are blurred by methodological deficiencies. Two studies showed a positive correlation between estimated male age and perfume quantity and complexity (Eltz et al., 1999, 2015). However, the proxy used to indicate age in these studies, wing wear, is known to be notoriously inaccurate. In orchid bees, the rate of wing wear accumulation can vary strongly between individuals (Eltz et al., 1999). In other insects, it has been shown that wing wear is correlated strongly with individual behavior, limiting the age-predictive power on a population level (Robbins, 1981; Foster and Cartar, 2011). The positive correlations between wing wear and perfume load found in previous studies on orchid bees were extremely noisy, including very low perfume loads in some seemingly very old individuals (Eltz et al., 1999, 2015), casting doubts on a general rule of linear perfume accumulation over time.

To obtain information about individual perfume development in orchid bees, we conducted a mark–recapture study with *Euglossa imperialis*, combining wing wear with a newly established age indicator quantifying the bleaching of the wing cell membrane. We correlated parameters of perfume load with estimated age. Furthermore, we manipulated the right hind-leg pouches of bees, sealing them with glue in order to ‘freeze’ the perfume status at first capture. By comparing perfume loads of left and right hind-leg pouches, we assessed the relative change of perfume in the time between first and second capture and correlated it with individual age. We looked at four different perfume traits that might potentially reflect male age or other fitness components: quantity, complexity, evenness of compound quantitative distribution, and volatility.

## MATERIALS AND METHODS

The experiments took place in March and April 2021 and 2022 at the La Gamba Research Station, Puntarenas, Golfo Dulce region, Costa Rica. This station is situated adjacent to the Parque Nacional Piedras Blancas in the southern Pacific region of the country. The study area experiences substantial annual precipitation (5.900 mm) and consistently warm temperatures, averaging 28°C (Huber et al., 2008). Male orchid bees [*Euglossa imperialis* (Cockerell 1922)] were attracted in the garden of the research station using chemical baits (1,8-cineole and methyl salicylate; Sigma-Aldrich, St Louis, MO, USA) between 07:30 and 12:00 h, when bees are most active. The chemicals were applied on filter papers, which were placed in tea strainers to prevent bees from direct access to the filter papers. We used *E. imperialis* as a model organism because it is a common species in the study area, relatively large and easy to handle, and can be attracted in large numbers to artificial baits (Roubik and Hanson, 2004). Further, *E. imperialis* was used in previous studies on orchid bee perfume biology (Eltz et al., 1999, 2019; Pokorny et al., 2017). The bees were caught with hand-nets and were individually marked with numbered plastic tags (Opalith-tags; Holtermann Imkereibedarf, Brockel, Germany). Furthermore, we took a photograph of the right forewing (see Age analysis) and sealed the right hind-leg pouch with cyanoacrylate glue (superglue, UHU). For this, we applied carefully multiple layers of glue directly on the shallow depression of the hind-leg pouch using a dissection needle (see Fig. S1). Previous analyses showed that right and left hind-leg pouches were found to contain very similar quantities of volatiles, suggesting that one body side represents a control for changes happening in the other (Eltz et al., 2019). After marking, sealing and photographing, the bees were set free. Marked bees were recaptured after a minimum of 3 days when appearing at chemical baits. Upon recapture, we took another photograph of the right forewing and visually checked the sealing of the right hind-leg. Individuals with intact hind-leg sealing were placed in Eppendorf caps, transferred to a freezer and both hind-legs were sampled on the same day (see Chemical analysis). Bees with visibly damaged sealing were discarded from further chemical analysis. To reduce stress of experimental individuals, newly captured males were either treated directly as described above or were transferred to darkened insectaries before treatment. Treatment times were kept as short as possible. Recaptured males were temporarily stored in a dark box before transferring them to a freezer.

### Age analysis

We used a digital, handheld microscope (HT-605, Shenzhen Hot Electronic Technology Co., Ltd, Shenzhen, China) and an LED light source (custom-made, Ruhr-Universität Bochum; see Fig. S2) for wing photographs. Both microscope and light source were modified with a microscope slide and the bee's right forewing was placed between slides. Furthermore, we placed reference filters (LEE filters, Pulheim, Germany) on the slide of the light source. The photos were analyzed using Photoshop Elements (v. 2022, Adobe, San Jose, CA, USA). To quantify wing transparency (bleaching), we calculated the ratio of the greyscale of the 2nd medial cell (Michener, 2007) and the reference filter. We took two photos per measurement and calculated the mean value. Furthermore, we quantified wing wear using a modified protocol (Mueller and Wolf-Mueller, 1993; Eltz et al., 1999). We counted the number of single nicks in the right forewing. However, in some cases, wings were extensively damaged. Therefore, we counted wing nicks and extensively damaged areas separately. We divided the wing margin into three areas, i.e. each wing had a maximum of three extensively damaged areas. We then multiplied the number of extensively damaged areas by three and added the number of nicks to determine the final value of wing wear. If the entire wing margin showed major excisions greater than the width of the distal submarginal cell (Mueller and Wolf-Mueller, 1993), we assigned the value of 3.5 as a maximum value for extensively damaged areas, resulting in a final value of wing wear of 10.5. However, there was no predetermined maximum value for single nicks, i.e. final values of 11 or higher were possible, but were not observed. We used this modified protocol in order to make this age indicator more independent of single, possibly high-impact events such as predator encounters (Eltz et al., 1999; Foster and Cartar, 2011).

To combine both age indicators, we standardized each value to percentage of the maximum and averaged the two percentages for each individual. We used a subset of bees (*N*=46) in 2021 to test the suitability of the new established age indicator. For that, differences of indicators between first and last capture were correlated with the number of days between first and last capture. These bees were repeatedly recaptured and were not sampled for chemical analysis (see below). For correlations with perfume traits at first and second capture, we used the respective age determined for first and second capture. For correlations analyzing the relative change in perfume traits, we used the mean value of the determined age at first and second capture.

### Chemical analysis

Hind-legs of bees were extracted separately by placing them in glass vials (Agilent Technologies, Santa Clara, CA, USA) containing 500 µl of *n*-hexane (Sigma-Aldrich). The *n*-hexane contained 1 mg l^−1^ 2-undecanone as internal standard (ISTD). To ensure dissolving of perfume contents in the solvent, all hind-legs were perforated with an insect needle before transferring them to the sampling vial. Samples were stored at −20°C until chemical analysis in Bochum, Germany. An HP 5890 II gas chromatograph coupled to an HP 5972 mass spectrometer (Hewlett-Packard, Palo Alto, CA, USA) was used for the analysis. The system was equipped with a DB-5MS column (30 m, 0.25 μm film thickness, 0.25 mm diameter) using splitless injection (1 μl). The temperature of the GC oven was programmed from 60 to 300°C at 10°C min^−1^ followed by 15 min isothermal at 300°C. For subsequent analysis, cuticular hydrocarbons, long-chain alcohols and acetates known to derive from bees' labial glands were analyzed separately (Eltz et al., 2005a; Pokorny et al., 2014, 2015b). We analyzed perfumes using the software ChemStation (Agilent Technologies). We identified compounds using commercial mass spectral libraries (Adams, 2001; NIST/EPA/NIH mass spectral database 2011) in conjunction with our own user libraries (see Table S1). To test the suitability of the superglue sealing, we analyzed the quantity [summed peak area (integrated ion currents) of all compounds] of compounds known to derive from bees' labial glands in the right and left hind-leg pouches (see Appendix). Further, we analyzed perfume complexity (number of compounds) and perfume quantity. To exclude bias resulting from variation in injection volume or detector sensitivity, we corrected compound abundances (peak areas) relative to the ISTD. Furthermore, we analyzed potential changes in volatility of perfume loads. For that, we calculated the slope of the regression lines between relative abundances of perfume compounds and their related retention times (RTs) for each sample. A positive slope indicates higher amounts of high molecular mass compounds with low volatility (high RT); a negative slope indicates higher amounts of more volatile (low RT) compounds. We excluded individuals containing only a single perfume compound (*N*=3) from this analysis. The use of a DB-5 GC column, in which apolar compounds elute approximately in order of molecular weight, allowed this approach. In addition, we analyzed the evenness of abundances (peak areas) of compounds in individual perfume blends. For that, we calculated Pielou's *J* (Pielou, 1967) for each sample using the Shannon index *H*′ (Eqn 1):
(1)

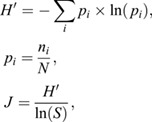
where *N* is the total amount (sum of peak areas) of all volatiles, *n_i_* is the peak area of a specific compound and *S* is the number of compounds per sample.

### Statistical analysis

**Fig. A1.**
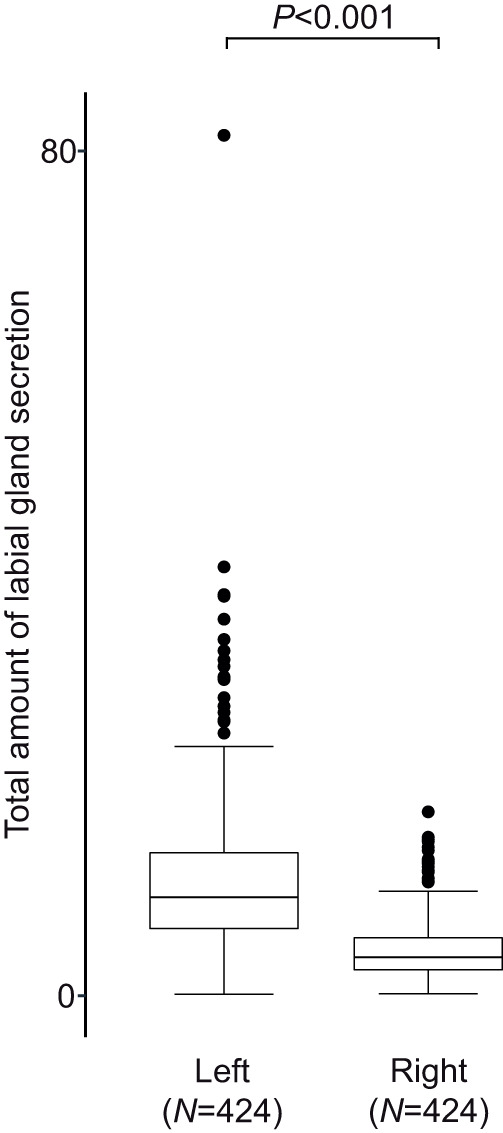
**Amount (sum of integrated ion currents) of compounds derived from bees' labial glands in left, non-manipulated hind-leg pouch and in right, manipulated (sealed) hind-leg pouch.** Amount is given as a ratio to the internal standard (ISTD). Boxplots show median (center line), upper and lower quartile (box limits), 1.5× interquartile range (whiskers) and outliers (black dots). Wilcoxon signed-rank test *P*-value and sample size (*N*) are shown.

To test whether the increase of the calculated age indicator of individual males was dependent on time between first and last capture, we conducted a linear regression analysis. We tested for differences in the total amount of compounds (sum of corrected integrated ion currents) deriving from bees' labial glands between right and left hind-leg pouches using the Wilcoxon signed-rank test (see Appendix, Fig. A1). We used non-parametric, two-sided Spearman rank correlations to test for effects of age (i.e. wing transparency combined with wing wear; see Age analysis) on perfume quantity, complexity, volatility and evenness. For visualization purposes, we fitted a non-linear trend line (LOESS: locally estimated scatterplot smoothing) using the geom_smooth function implemented in ggplot (R; v. 4.2.1) assuming an origin of zero for quantity and complexity. We analyzed both status of perfume traits of hind-leg pouches and the relative change (Δ) of perfume traits between first and second capture by subtracting right from left. We conducted all tests separately for the two study years (Table 1). Correlations were found to be broadly consistent between study years. Therefore, we combined both datasets for diagrams and pooled analyses. All statistical tests were done using SPSS (v. 28.0.1.1). The figures were plotted in R (v. 4.2.1).

**Table 1.**
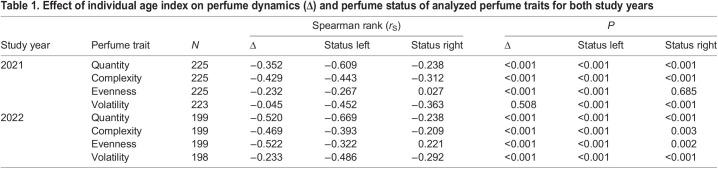
Effect of individual age index on perfume dynamics (Δ) and perfume status of analyzed perfume traits for both study years

## RESULTS

We marked 1318 individuals in the two study years. The overall resighting rate 1 day or later after marking was 41.7% (*N*=550). The recapture rate was 36.2% (*N*=477) 3 to 47 days after marking. A total of 424 individuals were used for chemical analysis. Bees used for testing the suitability of the age indicator (*N*=46) in 2021 were recaptured after 3 to 47 days (mean 18.9) after first capture. Bees used for perfume analysis were recaptured after an average of 8.0 days (min/max: 3/29) in 2021 and 7.7 days (3/24) in 2022.

### Age indicator

**Fig. 2.**
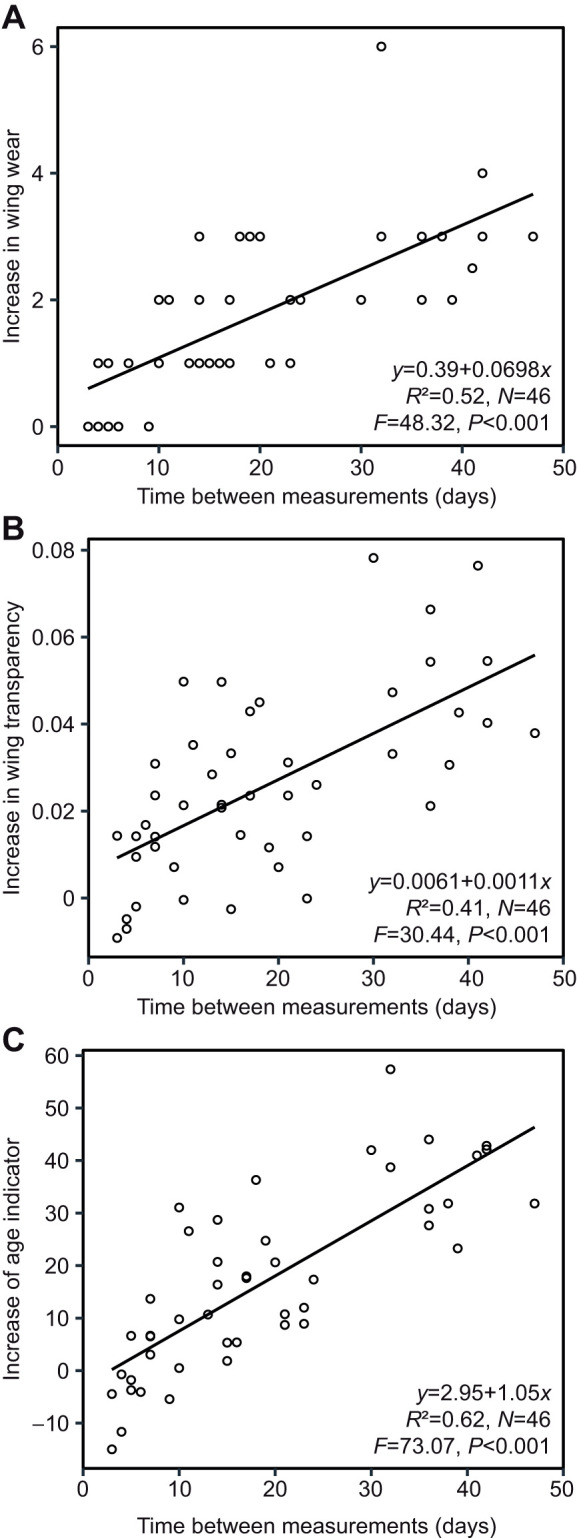
**Combined age indicator of wing wear and newly established wing transparency.** Dependency of the amount of increase in (A) wing wear, (B) wing transparency and (C) a combined factor of both variables (age indicator) on time between measurements in marked and recaptured *Euglossa imperialis*. Linear regression analysis.

Both the increase of wing wear and the increase of wing transparency were dependent on time between first and last measurements (linear regression, *r*²=0.52, *N*=46, *P*<0.001; Fig. 2A; linear regression, *r*²=0.41, *N*=46, *P*<0.001; Fig. 2B). The combined factor of both variables showed the strongest correlation with time and best linear fit (linear regression, *r*²=0.62, *N*=46, *P*<0.001; Fig. 2C), suggesting that it is suitable to determine at least roughly the individual age of the experimental bees.

### Correlates of perfume traits

In total, we found 209 different compounds present in the perfumes (see Table S1). The five most abundant compounds were 1,8-cineole (29.1% of the total amount of volatiles), germacrene D (18.1%), hexahydrofarnesyl acetone (14.4%), (*E*)-nerolidol (4.3%) and 2-(*E*),6-(*Z*)-farnesal (3.5%).

**Fig. 3.**
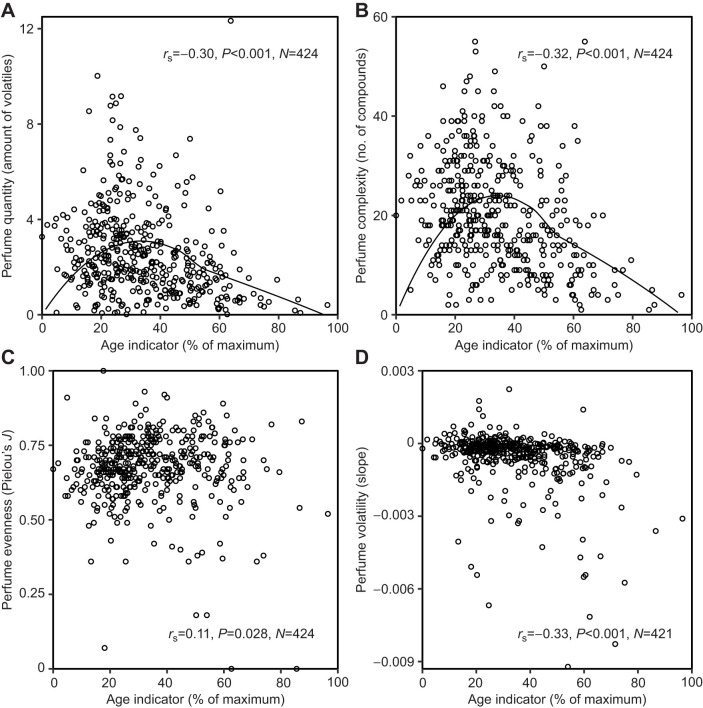
**Relationship between relative age and perfume traits at first capture.** (A) Perfume quantity, (B) complexity, (C) evenness and (D) volatility of the right, manipulated hind-leg pouch (time of first capture). (A) Amount is given as the ratio of summed peak area to the peak area of the internal standard (ISTD). (A–D) Spearman correlation coefficient (*r*_S_, two-sided), *P*-value and sample size (*N*) are shown. Age was assessed by combining measurements of wing wear and wing transparency (standardized to percentage of the maximum) at time of first capture. (A,B) Assuming that newly emerged individuals do not possess any perfume, a non-linear curve was fitted to visualize perfume development.

**Fig. 4.**
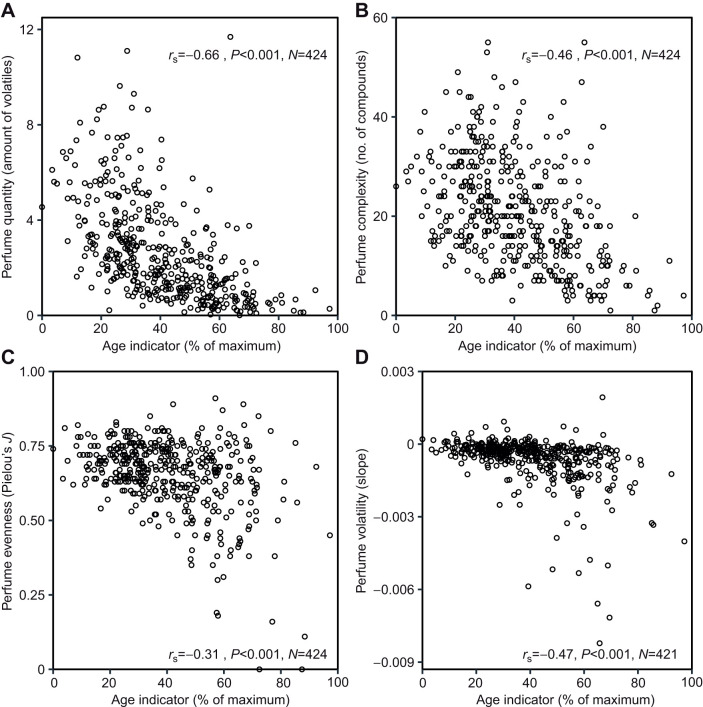
**Relationship between relative age and perfume traits at second capture.** (A) Perfume quantity, (B) complexity, (C) evenness and (D) volatility of the left, non-manipulated hind-leg pouch (time of second capture). (A) Amount is given as the ratio of summed peak area to the peak area of the ISTD. (A–D) Spearman correlation coefficient (*r*_S_, two-sided), *P*-value and sample size (*N*) are shown. Age was assessed by combining measurements of wing wear and wing transparency (standardized to percentage of the maximum) at time of second capture.

We found significant negative correlations of the age indicator at first capture with perfume quantity, complexity and volatility of the manipulated right hind-leg pouch (‘perfume status’ at first capture). Perfume quantity (*r*_S_=−0.30, *P*<0.001, *N*=424; Fig. 3A), perfume complexity (*r*_S_=−0.32, *P*<0.001, *N*=424; Fig. 3B) and volatility (*r*_S_=−0.33, *P*<0.001, *N*=421; Fig. 3D) showed a moderate negative correlation, whereas perfume evenness of compound abundances showed a weak, positive correlation (*r*_S_=0.11, *P*=0.028, *N*=424; Fig. 3C). When analyzing the left, non-manipulated hind-leg pouch in combination with the age indicator at second capture, we found significant negative correlations for all traits, with perfume quantity showing a strong correlation (Fig. 4A), whereas the other traits were moderately correlated (Fig. 4B–D).

Evidently, the sample of bees analyzed in this study showed a lack of newly emerged (completely empty) individuals. Therefore, to visualize perfume development including the very onset of perfume collection, we fitted a non-linear curve assuming zero perfume quantity/complexity at the minimum of the encountered range of the age indicator. This procedure depicts a peak of perfume quantity and complexity near the end of the first third of estimated male lifetime (Fig. 3A,B).

**Fig. 5.**
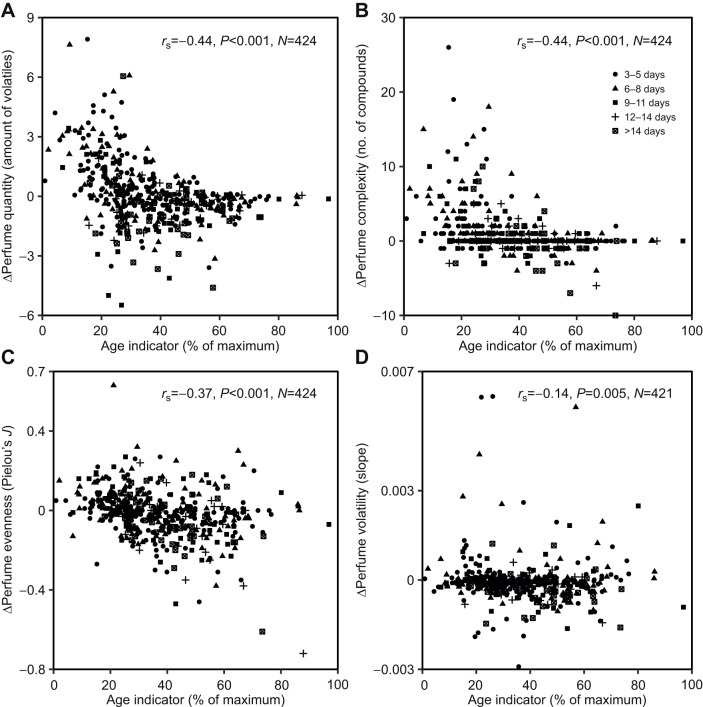
**Relationship between relative age and the relative change (Δ) of perfume traits between measurements.** (A) Perfume quantity, (B) complexity, (C) evenness and (D) volatility. (A) Amount is given as the ratio of summed peak area to the peak area of the ISTD. (A–D) Δ represents the difference between the non-manipulated left and the manipulated (sealed) right hind-leg pouch. Spearman correlation coefficient (*r*_S_, two-sided), *P*-value and sample size (*N*) are shown. Symbols indicate the time (days) between measurements. Age was assessed by calculating the mean value of the determined age at first and second capture.

To test whether the balance of uptake and exposure of perfume changes with age, we calculated the difference (Δ) of perfume traits between the left and right hind-leg pouch, representing the change in perfume between first and second capture. Negative values in quantity and complexity indicate more exposure than uptake (loss), positive values more uptake than exposure (gain). Of the 424 bees analyzed, 42.9% were recaptured after 3–5 days, 24.3% after 6–8 days, 13.4% after 9–11 days, 7.3% after 12–14 days and 12.0% after more than 14 days. We found significant negative correlations between the age indicator and Δ perfume quantity (*r*_S_=−0.44, *P*<0.001, *N*=424; Fig. 5A) and Δ perfume complexity (*r*_S_=−0.44, *P*<0.001, *N*=424; Fig. 5B), suggesting that perfume balance was exposure-biased in all but the youngest bees. Further, we found weaker, but significant negative correlations with estimated age for Δ perfume compound evenness (*r*_S_=−0.37, *P*<0.001, *N*=424; Fig. 5C) and Δ compound volatility (*r*_S_=−0.14, *P*=0.005, *N*=421; Fig. 5D).

## DISCUSSION

Male orchid bees visit a wide range of different scent sources to concoct complex perfume blends that are broadly species-specific (Weber et al., 2016). The cumulative way of euglossine perfume making appears to be in accordance with the idea of perfumes being honest indicators of age, i.e. survival (Eltz et al., 1999). To evaluate the plausibility of that idea our study measured how perfume traits develop over a bee's lifetime, using an improved age estimate.

In contrast to previous studies with other species (*Euglossa cognata* in Panama: Eltz et al., 1999; and *E. dilemma* and *E. viridissima* on the Yucatán peninsula, Mexico: Eltz et al., 2015), the results of the present study weaken the idea of a cumulative increase of perfume loads. None of the analyzed perfume traits correlated positively with estimated age of male *E. imperialis*, at least not when the entire life span/span of wing degradation was considered. On the contrary, we found a decrease in perfume quantity and complexity with age. Male euglossines start with zero perfume in their pouches when emerging (Henske et al., 2023). This suggests that the youngest individuals in our sample must have been disproportionately active/successful in volatile collection, whereas in the majority of middle-aged and older bees, the balance was skewed towards perfume expenditure, resulting in ever more depleted perfume stores. In the following, we will discuss the potential mechanisms that could have contributed to this pattern of perfume dynamics.

Age-related shifts in the behaviors resulting in perfume uptake and exposure could be responsible for the observed patterns. Previous observations suggest that volatile collection and exposure (display) are alternating, not mutually exclusive activities. Mark–recapture studies showed that both young and old bees collect volatiles at artificial chemical baits (Ackerman and Montalvo, 1985; Stern, 1991; Eltz et al., 1999). Equally, courtship display has been observed in individuals of widely differing age. Caged males of *E. dilemma* started to display within days after emergence, and no age-dependent changes in display activity were observed over the course of mating experiments (Henske et al., 2023). In addition, in *Eulaema meriana* (Stern, 1991), the same individuals could be observed repeatedly at natural display sites for up to 49 days. Thus, male orchid bees engage in both collection and exposure of volatiles over much of their lifetime. However, the time allocated to the respective behaviors could certainly vary with age, as could the efficiency by which the behaviors are performed. Conceivably, the efficiency of detecting elusive volatile sources could decrease in aging individuals, leading to the observed decline of perfume load with estimated age. It is known from studies in other insects that the sensitivity of the antennae for specific stimuli decreases with age (Rees, 1970; Seabrook et al., 1987; Otter et al., 1991; He et al., 2017); this has also been shown in honey bees (Vetter and Visscher, 1997). In orchid bees, a decline in sensitivity could lead to older males no longer being able to efficiently localize natural volatile sources, tipping the balance towards exposure and resulting in declining loads. Alternatively, male physical strength could decrease with age. The present and previous studies show that wings become more and more damaged with increasing age (Eltz et al., 1999, 2015) and the visit of scent sources might become more difficult for older individuals.

Choosing from the alternative scenarios depicted in Fig. 1, perfume development in *E. imperialis* follows a peaking curve, with the climax of perfume load reached rather early in life. Although Fig. 3 does not clearly show a well-defined peak, such a shape is the necessary conclusion given that perfume load must start from zero in freshly emerged individuals (Henske et al., 2023). A range of factors may have contributed to blurring the picture. First, our sample appears to have included few very young (empty) individuals at first capture in both years. Nevertheless, an overrepresentation of small perfume loads in the sealed right hind-leg of young males is visible (Fig. 3) as compared with perfume loads in the non-manipulated left hind-leg at second capture (Fig. 4), at a point when males were 3 to 29 days (mean±s.d.: 7.8±5.0 days) older and had had the chance to collect additional volatiles. Second, the age indicator used in our study provides only a crude estimate of age, sufficient to distinguish young from old bees, but not accurate enough to discern smaller age differences (see below). Third, there is likely substantial variability in perfume development between individual males, further inflating the noise in relationships between age and perfume parameters.

It remains to be seen whether the early peak of perfume development found in *E. imperialis* is exceptional or whether it represents a more general pattern in male euglossines. Two previous studies on other orchid bee species showed overall positive correlations of perfume quantity and complexity with wing wear (Eltz et al., 1999, 2015). However, like in the present study, correlations were very noisy, and, in both studies, there were seemingly old individuals (with large numbers of wing nicks) that had very little perfume. Thus, the possibility of a peaking relationship between age and perfume load had already been discussed (Eltz et al., 1999, 2015). The larger sample size in the present study, along with the combination of two independent age indicators and the replication of the study in two different years, certainly corroborates a peaking relationship.

Changes in wing transparency with age are probably due to UV-induced degradation of cuticle pigments (‘photobleaching’; Koch et al., 2014) and may therefore be less affected by differences in individual behavior in comparison to traditional wing wear (Robbins, 1981; Eltz et al., 1999; Foster and Cartar, 2011). However, in measurements of wing transparency there was also substantial variability that was not accounted by time between measurements. Furthermore, owing to the difficulty of finding nests, we do not know about the variability of wing transparency among freshly hatched male euglossine bees. Clearly, the power of accurately predicting the age of an individual male orchid bee is still very limited. Additional noise in our data may have derived from imperfections in ‘freezing’ the perfume status of males by sealing one pouch at first capture. In general, left and right hind-leg pouches of individual *E. imperialis* do contain almost identical quantity and composition of volatiles, but differences can occur (Eltz et al., 2019). Nevertheless, the superglue barrier efficiently prevented volatile uptake and exposure (see Appendix, Fig. A1). Further, the major compounds of perfume loads we found in this study are congruent with previous findings in *E. imperialis* (Pokorny et al., 2017; Darragh et al., 2023).

A traditional hypothesis of age and sexual selection predicts that females should prefer older males because they have proven their ability to survive (Brooks and Kemp, 2001). Most support for this hypothesis comes from mammal and bird species (Brooks and Kemp, 2001), whereas evidence from insects is rare. In previous studies on crickets (*Gryllus* spp.), females preferred older males, which were less parasitized and therefore produced more spermatophores (Zuk, 1988; Simmons and Zuk, 1992). In contrast, there is ample evidence for reproductive senescence (Lemaître and Gaillard, 2017), the decrease in reproductive success with increasing age, in vertebrates (Nussey et al., 2013), reptiles (Massot et al., 2011), fish (Morbey et al., 2005) and invertebrates (Zajitschek et al., 2009). In insects, the selection of younger males, possibly owing to reproductive senescence, was shown in the lekking sandfly *Lutzomyia longipalpis*: females that chose middle-aged males raised more offspring. Older males represented poorer quality males, perhaps owing to a decline in sperm quality or sperm transfer ability (Jones et al., 2000). In *Drosophila melanogaster*, the reduction in larval viability of offspring raised by older males indicates that older fathers may produce offspring of inferior genetic quality (Price and Hansen, 1998), indicating a trade-off between traits that increase the ability to survive and traits that favor reproduction success (Hansen and Price, 1995). There appears to be no straightforward relationship between age and genetic quality of an individual, and the complex relation between sex, mortality and ageing is still poorly understood (Bonduriansky et al., 2008). In orchid bees, there might be a trade-off in males between the demonstration of youth (e.g. sensory abilities) and the demonstration of foraging history and survival. Such a trade-off could partly explain the substantial individual variability in perfume accumulation in male orchid bees. Females choosing males with strong perfume signals may enhance the speed of offspring foraging rather than overall lifetime yield.

### Conclusions

Although perfumes of male orchid bees most likely evolve by sexual selection (indicator function) in addition to natural selection (recognition function), their exact informative content remains elusive. Perfumes do not linearly reflect age and survival in *E. imperialis*, but may encode other fitness components such as sensory acuteness or orientation skills. In orchid bees, a trade-off between cumulative and non-cumulative fitness components may have led to variable and diverging results in studies of perfume dynamics.

## APPENDIX

### Super-glue sealing of hind-tibial pouches works: background and evidence from labial gland lipids

During volatile collection, male bees apply mixtures of long-chain aliphatic compounds (‘lipids’) from labial glands on the odoriferous surface (Whitten et al., 1989). The bees then absorb the resulting blend of gland secretion and volatiles dissolved in it, using fore-tarsal brushes to transfer it to the specialized pouches on the hind tibiae (Vogel, 1966; Kimsey, 1984). These pouches are cuticle invaginations with a sponge-like inner structure possessing a shallow depression on the surface functioning as an interface for volatile uptake and exposure (Eltz et al., 2005b). Capillary forces lead to volatile uptake (Vogel, 1966), resulting in the deposition of a blend of exogenous fragrance compounds and endogenous labial gland lipids (Williams and Whitten, 1983; Whitten et al., 1989). The labial gland lipids are then selectively reabsorbed from the pouches and are transferred back to the cephalic labial glands for reuse during volatile collection (Eltz et al., 2007).

We tested for differences in the total amount of compounds deriving from bees' labial glands between the right (sealed) and left (non-manipulated) hind-leg pouch. Left hind-leg pouches contained significantly more labial gland compounds (mean±s.d.: 231.8±365.4%) than manipulated right hind-leg pouches (Wilcoxon, *P*<0.001, *N*=424; Fig. A1). This is in accordance with reabsorption of labial gland compounds from hind-leg pouches (Eltz et al., 2007). It further shows that, owing to the super-glue barrier, the deposition of new labial gland lipids (and dissolved volatiles) was at least severely reduced in the sealed right hind-leg. It also suggests that the super-glue sealing effectively prevented the exposure of perfume from the right hind-leg during display.

## Supplementary Material

10.1242/jexbio.246995_sup1 Supplementary information
