# Supplementary material for: Age-dependent perfume development in male orchid bees, Euglossa imperialis
Source: J Exp Biol. 2024 Mar 22;227(6):jeb246995. doi: 10.1242/jeb.246995 (PMC11006377; doi:10.1242/jeb.246995)
Supplement: Supplementary information [file jexbio-227-246995-s1.pdf]

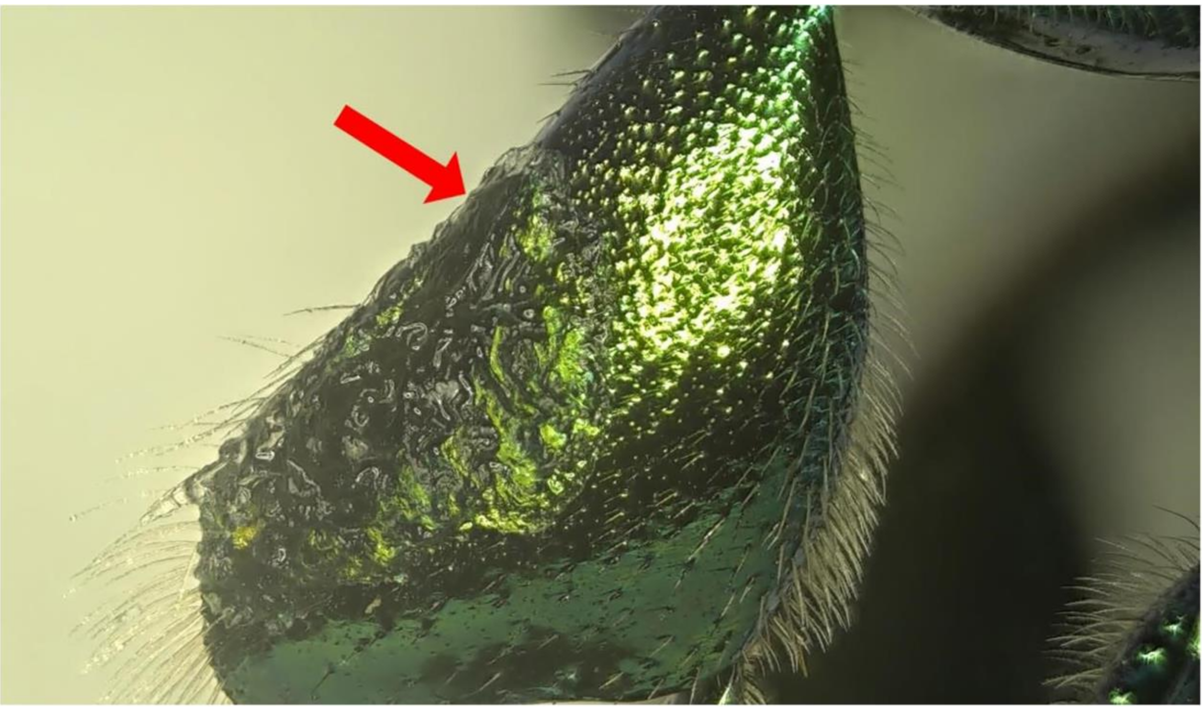

Fig. S1. Right hind-leg pouch of *Euglossa imperialis* sealed with cyanoacrylate superglue. Red arrow indicates the position of the interface for volatile uptake and exposure. Photo by Janosch Dohrs.

Journal of Experimental Biology • Supplementary information

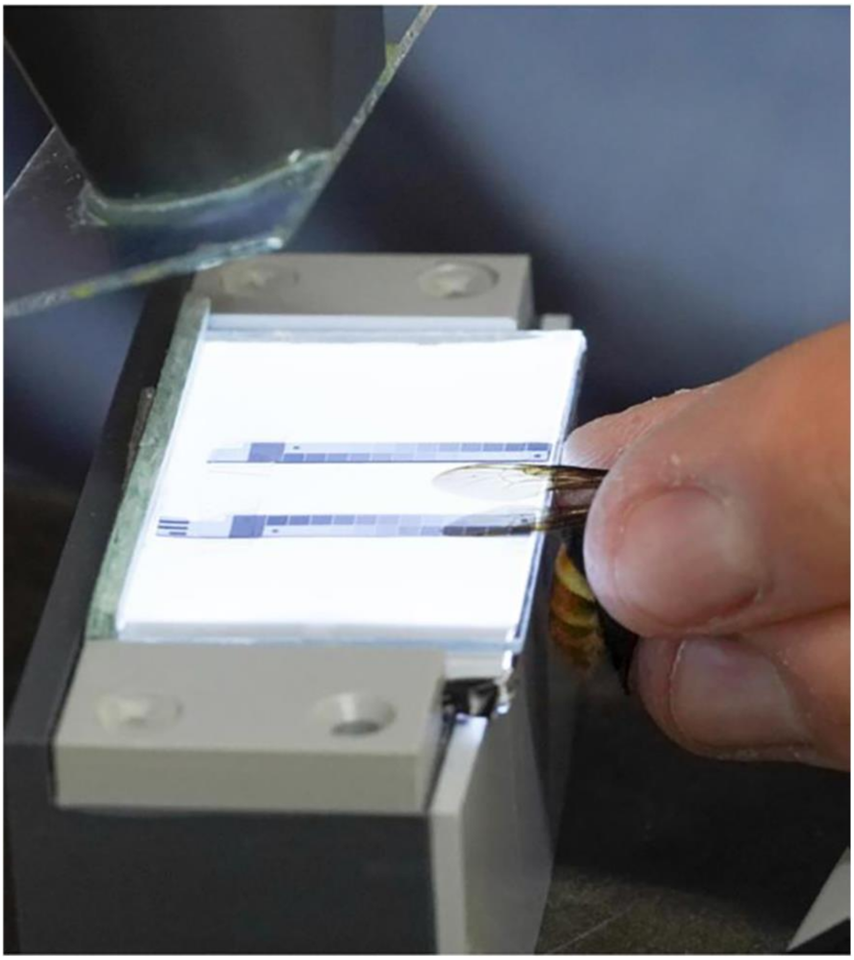

Fig. S2. Digital microscope and LED light source (with grey scale) for quantification of wing transparency (bleaching) as an age indicator. Photo by Thomas Eltz.

Journal of Experimental Biology • Supplementary information

Table S1. Composition of perfumes in the left, non-manipulated hind-leg of male *Euglossa imperialis* (*N*=424) in La Gamba, Costa Rica. A total of 209 different compounds were found. The table shows the compounds (*N*=60) that were found in at least 5% of the sampled individuals. Compounds were identified by comparing spectra and retention indices with those in commercial mass spectral libraries[1] and/or of synthetic standards (*). The ten most abundant ions are given for compounds that could not be identified. The Kovats retention index is given as "RI", the average relative peak area of a compound is given as "Rel. abundance" (perfume compounds only), the proportion of individuals possessing a specific compound is given as "% No. Ind.". Compound ID refers to the raw data table provided in data repository (see Data availability).

Compound name / Mass spectra data	RI	Rel. abund	% No. Ind.	ID	Data base
1,8-cineole	1033	37.82	99.76	5	*, Adams
Hexahydrofarnesyl acetone	1842	13.74	99.06	32	*, NIST
Germacrene D	1495	16.17	98.82	19	*, Adams
(*E*)-Caryophyllene	1422	2.36	88.21	67	Adams
(*E*)-Nerolidol	1564	3.39	85.38	24	*, Adams
α-Pinene	938	2.29	84.67	1	*, Adams
43,55,93,81,67,105,135,107,69,91	2225	1.64	84.20	38	n/a
2-(*E*),6-(*Z*)-Farnesal	1709	2.78	81.60	27	Adams
α-Humulene	1466	0.59	72.64	69	Adams
Bicyclogermacrene	1494	0.67	66.98	21	Adams
2-(*E*),6-(*E*)-Farnesal	1740	1.67	65.09	29	Adams
α-Phellandrene	1000	2.89	62.97	64	Adams
Limonene	1028	1.24	50.24	4	*, Adams
β-Elemene	1398	0.28	50.00	13	Adams
β-Pinene	984	0.35	48.58	63	*, Adams
δ-Amorphene	1518	0.27	47.88	23	Adams
43,55,93,81,67,107,69,91,53,44	1797	0.56	47.41	71	n/a
β-Copaene	1434	0.23	44.81	15	Adams
55,82,67,81,96,95,69,43,68,54	2362	0.56	43.63	41	n/a
β-Bisabolene	1502	0.66	42.45	22	*, NIST
Methyl salicylate	1184	0.75	41.27	8	*, Adams
α-Copaene	1385	0.22	37.97	12	Adams
Ethyl salicylate	1277	1.73	37.50	10	Adams
93,91,79,105,67,107,55,81,43,95	1719	0.52	32.78	202	n/a
43,81,93,55,67,105,68,107,135,69	2658	0.57	32.31	48	n/a

Journal of Experimental Biology • Supplementary information

Compound name / Mass spectra data	RI	Rel. abund	% No. Ind.	ID	Data base
Sabinene	973	0.23	32.08	87	Adams
43,91,93,79,105,107,55,119,159,77	1593	0.14	30.66	25	n/a
δ-3-Carene	1001	1.26	28.54	85	Adams
43,161,69,91,93,105,81,79,77,55	1450	0.11	26.18	68	n/a
γ-Muurolene	1475	0.32	22.88	110	NIST
67,79,55,81,80,85,93,95,54,43	2290	0.25	21.93	39	n/a
43,69,119,93,79,80,81,91,59,55	1609	0.15	20.05	70	n/a
(*E*)-Farnesene epoxide	1617	0.14	19.10	86	NIST
43,69,55,81,67,83,57,44,93,82	1722	0.18	16.27	28	n/a
α-Thujene	921	0.10	16.04	102	Adams
Myrcene	987	0.14	15.33	108	Adams
161,91,81,43,105,93,79,77,119,67	1452	0.03	14.86	94	n/a
Terpinen-4-ol	1189	0.10	11.79	7	*, Adams
69,81,43,67,55,93,109,121,79,53	1712	0.05	10.85	193	n/a
43,55,107,81,123,91,121,79,93,109	1868	0.05	10.85	218	n/a
121,93,43,58,107,79,91,77,67,94	1325	0.03	10.38	93	n/a
43,69,55,95,71,83,56,109,82,123	1874	0.04	10.14	103	n/a
Terpinolene	1086	0.14	10.14	109	Adams
Caryophyllene oxide	1591	0.04	9.91	95	Adams
α-Terpineol	1200	0.12	9.91	107	Adams
95,105,120,189,107,55,79,81,67,91	1974	0.04	8.96	82	n/a
43,120,92,56,152,70,61,121,55,57	1194	0.08	8.73	9	n/a
(Z,Z)-2,6-Farnesol	1696	0.07	8.02	237	NIST
γ-Amorphene	1501	0.03	7.08	159	Adams
93,59,43,79,119,105,67,91,81,80	1648	0.03	6.84	150	n/a
69,93,43,71,68,81,80,67,107,55	1977	0.12	6.84	187	n/a
71,99,43,58,55,57,85,59,72,42	1169	0.02	6.60	254	n/a
Decyl acetate	1389	0.05	6.37	120	Adams
Zingerone	1648	0.02	6.37	128	NIST
43,57,55,97,69,56,70,71,83,111	1946	0.03	6.37	165	n/a
69,81,55,67,109,95,43,68,136,123	2205	0.05	6.13	90	n/a
159,43,91,105,177,81,117,93,55,67	1656	0.02	6.13	215	n/a
93,91,77,136,92,79,121,43,105,94	1055	0.15	5.90	106	n/a
159,43,91,93,105,220,55,79,67,133	1691	0.02	5.90	216	n/a
69,109,67,123,55,43,179,81,53,93	1609	0.07	5.42	140	n/a

[1]Adams (2001). Identification of Essential Oil Components by Gas Chromatography/ Quadrupole Mass Spectroscopy. Carol Stream, USA: Allured Publishing Corporation; NIST/EPA/NIH mass spectral database 2011
